# Binding-Site Assessment by Virtual Fragment Screening

**DOI:** 10.1371/journal.pone.0010109

**Published:** 2010-04-09

**Authors:** Niu Huang, Matthew P. Jacobson

**Affiliations:** 1 National Institute of Biological Sciences, Beijing, Beijing, China; 2 Department of Pharmaceutical Chemistry, University of California San Francisco, San Francisco, California, United States of America; German Cancer Research Center, Germany

## Abstract

The accurate prediction of protein druggability (propensity to bind high-affinity drug-like small molecules) would greatly benefit the fields of chemical genomics and drug discovery. We have developed a novel approach to quantitatively assess protein druggability by computationally screening a fragment-like compound library. In analogy to NMR-based fragment screening, we dock ∼11000 fragments against a given binding site and compute a computational hit rate based on the fraction of molecules that exceed an empirically chosen score cutoff. We perform a large-scale evaluation of the approach on four datasets, totaling 152 binding sites. We demonstrate that computed hit rates correlate with hit rates measured experimentally in a previously published NMR-based screening method. Secondly, we show that the *in silico* fragment screening method can be used to distinguish known druggable and non-druggable targets, including both enzymes and protein-protein interaction sites. Finally, we explore the sensitivity of the results to different receptor conformations, including flexible protein-protein interaction sites. Besides its original aim to assess druggability of different protein targets, this method could be used to identifying druggable conformations of flexible binding site for lead discovery, and suggesting strategies for growing or joining initial fragment hits to obtain more potent inhibitors.

## Introduction

Since the completion of the human genome, there has been much interest in the “druggability” of new potential drug targets, and what fraction of the proteome is druggable. In this paper we are concerned with protein druggability in the sense defined by Hopkins and Groom [1], i.e., the ability of a protein to bind small, drug-like molecules with high affinity. For many classes of protein binding sites, such as the ATP binding sites in kinases, there is little ambiguity about whether the site is druggable; the challenge in developing inhibitors in such cases is achieving selectivity and other desired properties. However, not all biological targets are druggable since only certain binding sites are complementary to drug-like compounds in terms of physicochemical properties (i.e. size, shape, polar interactions and hydrophobicity) [1], [2]. An accurate method for predicting druggability would be particularly valuable for assessing emerging classes of binding sites such as protein-protein interactions (PPI) [3] and allosteric sites [4], which are generally considered more challenging but are attracting increasing interest in both academia and industry as drug targets. For example, while some PPI sites have led to potent small molecule inhibitors, others have not despite substantial effort [5], [6].

A first step in evaluating target druggability is to detect the presence of binding pockets of suitable size, shape, and composition to accommodate drug-like molecules. Many such methods have been developed and tested using training sets of ligand binding sites extracted from the Protein Data Bank (PDB). Several in-depth reviews are available that summarize computational methods for protein binding pocket detection [7], [8], [9], many of which can be classified as geometry-based [10], [11], [12], [13], information-based [14], [15] and energy-based algorithms [16], [17]. Combinations of these strategies have also been developed [18], [19], [20], [21], [22]. In addition, more complex free-energy calculation methods have also been used to predict binding sites and identify energetically favorable binding site residues, including computational solvent mapping [23] and grand canonical Monte Carlo simulations [24].

The presence of a “suitable” protein pocket is necessary but not sufficient to guarantee potent binding of drug-like small molecules. A few studies have attempted to more directly predict druggability of binding sites. Several studies have predicted protein druggability on the basis of sequence and structural homology to known drug targets [1], [2], [8]. However, not all members of the same protein family are equally druggable [25]. More importantly, such methods cannot be used to assess druggability of novel target families. Recently, an alternative approach was described to predict the maximal affinity for a passively absorbed oral drug to a given binding site, by quantitatively approximating the physical forces driving protein-ligand binding. Specifically, hydrophobic surface area and curvature of the binding pocket were used to fit the binding affinities of a training set of protein-ligand binding complexes. Notably, this model was successfully applied to predict the relative druggability of two novel targets before experimental validation [26].

To date, the most extensive experimental assessment of druggability on various targets has been performed by Hajduk and coworkers [27]. The heteronuclear-NMR-based technique was applied to screen fragment-like libraries against a set of 23 protein targets containing 28 different binding sites. This study revealed that small ligands bind almost exclusively to well-defined binding pockets on the protein surface, independent of their affinity. Remarkably, the authors observed a strong correlation between the experimental NMR hit rate and the ability to bind drug-like ligands with high affinity in a particular binding site. Furthermore, they derived a linear regression model to fit the experimentally measured hit rates to physicochemical descriptors of these 28 binding pockets. These results suggested that the druggability of a particular binding site is related to its propensity to bind low-affinity, fragment-size compounds.

We wondered whether *in silico* fragment screening would also be useful in this regard, with the obvious advantages of speed and cost relative to experimental screening. Here we describe the development and evaluation of such a method, making use of a molecular mechanics-based scoring method for the protein-ligand interactions (Method Section) [28], [29]. Specifically, we report the results of virtually screening ∼11,000 diverse fragment-like compounds against a total of 152 protein binding sites, including the training dataset and external dataset studied by Hajduk and coworkers [27]. We demonstrate that the hit rate calculated from computationally screening a diverse fragment library correlates with the hit rate measured experimentally from the NMR-based screening method, despite the fact that we could not directly replicate the experiment *in silico* because the fragment libraries used for the NMR screening are proprietary. Secondly, we show that the *in silico* fragment screening method can be used to distinguish known druggable and non-druggable targets, including both enzymes and protein-protein interaction sites. Finally, we explore the sensitivity of the results to different receptor conformations, including flexible protein-protein interaction sites.

## Results and Discussion

### Comparison between NMR-based and Virtual Fragment Screening

**Figure 1:**
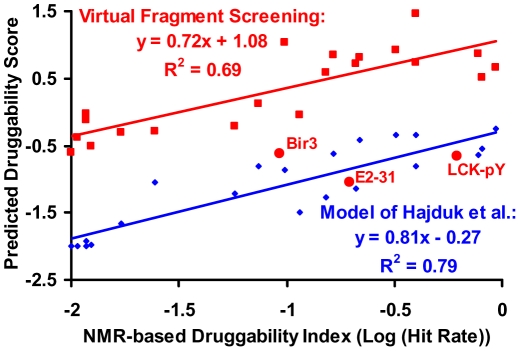
Correlation between the experimental NMR hit rate and our calculated druggability score (red line) for 21 binding sites as described in the text, comparing to the Hajduk et al. predictive model for these 21 sites (blue line). Note that three outliers in our druggability calculation were excluded in regression analysis, and are only labelled here for visualization purpose.

The key results of virtual fragment screening against the Hajduk et al. training dataset [27] are summarized in Table 1 and Figure S1. Table 1 summarizes the druggability scores measured from NMR-based screening, predicted by an empirically fitted model by Hajduk et al., and predicted by our virtual fragment screening method. Our calculated druggability scores correlate reasonably well with the NMR-based fragment screening results (Figure 1) except for three binding sites (Bir3, E2–31 DNA site, and LCK pTyr binding site), for which we compute much lower hit rates. Our primary goal is not to reproduce the NMR screening data per se, but to predict druggability, as we discuss in the next section. Nonetheless, these outliers deserve brief comment. First, the experimentally measured binding affinity of the best fragment hits ranges are rather weak, from 200 to 1,000 µM, for these three outliers, several-fold higher than other targets with similar NMR hit rates (less than 50 µM) [27]. Further, although these sites were classified as druggable or moderately druggable based on the NMR hit rates, no high-affinity druglike binders have been reported to our knowledge. The LCK pTyr binding site and Bir3 have been suggested to be not druggable due to their highly polar or very small binding sites [30]. The reported LCK pTyr site “druglike” ligand contains a diphosphonophenylalanine group to target the pTyr site, and a cyclohexane ring inserts deeply into an extra spatially distinct hydrophobic binding pocket (pTyr+3) [31], which further indicates that LCK pTyr site is not a truly druggable site by itself. We cannot rule out the possibility that these outliers reflect a deficiency in our method, but for the reasons discussed above, we have excluded them from further analysis.

**Table 1:** Targets, binding sites, and hit rate data from NMR-based fragment screening and two different computational models.

Target	Binding site	PDB ID	NMR-based Screening^a^Log (Hit Rate)	Model of Hajduk et al.^a^	Virtual Fragment ScreeningLog (Hit Rate)
AK	Adenosine	1lii	−0.66	−0.42	0.82
Akt-PH	IP3	1h10	−1.91	−1.98	−0.51
Bcl-X_L_	Bak	1bxl (1ysn)c,d	−0.11	−0.64	0.86 (0.88)
Bir3^b^	Peptide	1g3f	−1.03	−0.72	−0.62
CMPK	CMP	1q3t^c^	−1.13	−0.72	0.12
E2-31^b^	DNA	1dhm^c^	−0.71	−0.72	−1.05
ErmAM	SAH	1qam	−1.01	−0.87	1.04
FBP	DNA	1j4w	−1.61	−1.04	−0.28
FKBP	FK506	1fkj	−0.03	−0.24	0.66
FKBP	2nd site	1fkj	−1.24	−1.22	−0.22
HI-0065	ADP	1fl9	−0.82	−1.28	0.59
LCK^b^	pTyr	1lkl	−0.21	−1.07	−0.67
LFA	IDAS	1rd4	−0.40	−0.35	0.74
MDM2	P53	1rv1 (1ycr)^d^	−0.49	−0.35	0.92 (0.45)
MurI	Glu	1zuw	−1.93	−2.00	−0.12
PAK4	ATP	2cdz^c^	−0.78	−0.63	0.85
PDZ-PSD95	Peptide	1iu0	−2.00	−1.99	−0.60
Pin1	Peptide	1i8h	−0.94	−1.49	−0.05
PTP1B	Catalytic pTyr	1ph0	−0.68	−1.15	0.72
PTP1B	Noncatalytic pTyr	1ph0	−1.77	−1.66	−0.31
SARS N-term	RNA	1ssk^c^	−1.93	−1.92	−0.03
SCD	Substrate	1g4k	−0.09	−0.55	0.51
Survivin	Bir3	1e31	−1.97	−1.99	−0.37
UK	Peptide	1fv9	−0.40	−0.81	1.46

For the NMR-based screening results and the predictive model of Hajduk et al., druggable is defined as log (Hit Rate) >−1.0 and non-druggable as log (Hit Rate) ≤−1.0; the corresponding cut-off in our virtual fragment screening model is 0.36.

a from Reference [27].

b Three outliners (Bir3, E2–31 DNA site and LCK pTyr binding site) identified in our study.

c Structures were not reported in the Hajduk et al. dataset.

d For Bcl-xl and MDM2, we used two structures, peptide-bound (1bxl and 1ycr, respectively) and small ligand bound (1ysn and 1rv1, respectively).

Figure 1 presents the correlation between the NMR-based druggability score and our calculated druggability score for the remaining 21 binding sites. Encouragingly, a reasonable correlation is achieved (R^2^ = 0.69), especially considering that the compounds screened by NMR and by docking are different. The empirical model developed by Hajduk et al. by fitting to this data, using multiple adjustable parameters, gives a correlation of R^2^ = 0.79 for these 21 binding sites, and the slopes of the two models are similar in Figure 1 (0.72 and 0.81, respectively).

Hajduk et al. defined binding sites as “highly druggable” if they have a log(hit rate)>−1.0. Based on the correlation in Figure 1, the corresponding value of the computational log(hit rate) is 0.36, and we use this value to classify proteins as druggable or non-druggable in the following sections. Note that, although Hajduk et al. distinguish between “highly druggable” and “moderately druggable”, we use a simple binary classification for simplicity.

### Classification of Binding Sites as Druggable/Non-Druggable

Using the druggability score cutoffs derived above, we evaluated the ability of the virtual fragment screening protocol to classify the binding sites in the Hajduk et al. external dataset, which contains 72 targets, including 35 classified as druggable and 37 as non-druggable. Table S1 summarizes the druggability scores calculated using the empirical model of Hajduk et al. and our virtual fragment screening method.

In evaluating the success of our method, we put more emphasis on “true positives”, i.e., the ability to identify proteins as druggable when they have in fact been shown to bind small drug-like molecules with high affinity. By contrast, the lack of a published, potent small molecule inhibitor does not necessarily prove that a target is not druggable, and thus we put less emphasis on putative “false positives”. In addition, in practical application, incorrectly predicting a site to be non-druggable when it is in fact druggable arguably would be worse than incorrectly predicting a non-druggable site to be druggable. However, it is clear from the ROC plot that a different hit rate cutoff could also be used to reduce the number of false positives while maintaining a relatively high true positive rate, if desired.

**Figure 2:**
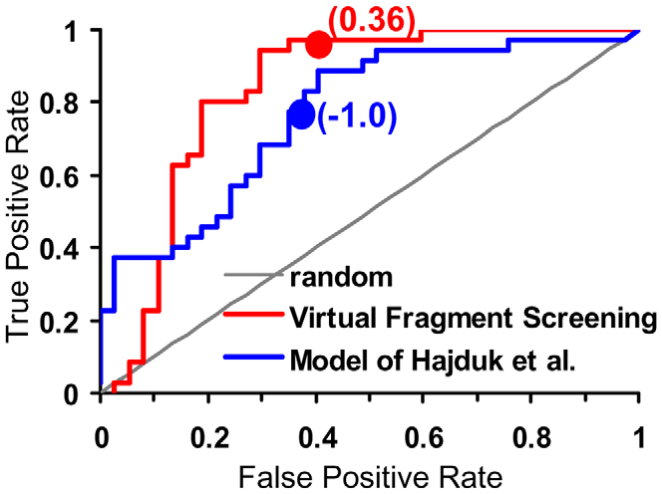
ROC curve plotting the false positive rate vs the true positive rate as a function of the score used for differentiating druggable vs non-druggable binding sites in the external dataset of Hajduk et al. The values for the default cutoff scores are marked with a solid circle and shown in parentheses. For this analysis, true positives were defined as the 35 binding sites with known high affinity ligands, while true negatives were the remaining 37 binding sites.

As shown in Figure 2, our method is able to effectively distinguish between druggable and non-druggable sites. Encouragingly, our method, using the default hit rate cutoff, correctly identified almost all the true positives except for only one case, protein kinase C (PKC-delta). Because PKC–ligand binding was shown to be dependent on phospholipid binding [32], it is perhaps not surprising that our method failed to predict PKC-delta as a druggable site since we do not treat the effects of membrane binding. With respect to false positives, we classified several proteins that bind sugars or sugar analogs as moderately druggable, whereas these are annotated as non-druggable targets due to the lack of reported high-affinity druglike binders in the literature.

Based on a survey of recent literature, a few of the putative “false positives” are possibly incorrectly classified as non-druggable (Table S1). For example, fatty acid binding protein (FABP) was annotated as a non-druggable target; however, both ours and the empirical model of Hajduk et al. predicted it to be highly druggable. Small fragment-like ligands were identified by an NMR-based screening method [33], and also a high-affinity drug-like inhibitor was recently reported to be an effective therapeutic agent to prevent and treat metabolic diseases [34]. In contrast to prediction of Hajduk et al., our method ranked three targets (guanylate kinase, guanine nucleotide-binding protein, and deoxynucleoside-monophosphate-kinase) as highly druggable hits, whereas they were annotated as non-druggable targets. Considering the similarity of these targets to well-known kinase targets, we wonder whether these three nucleoside/nucleotide binding proteins may prove to be druggable.

### Application to Known Drug Targets with Multiple Conformations

As an additional data set, we assembled a diverse set of well-known drug targets that have multiple crystal complex structures available, and generally display significant sidechain movement upon binding to different ligands [35] (Table 2). Thus, these targets serve as additional positive controls, and also allow us to investigate the consequences of protein conformational flexibility.

**Table 2:** Druggability score calculated on 15 well-known drug targets.

Drug Target	PDB ID	RMSD_ave_ (Å)	RMSD_max_ (Å)	Log (Hit Rate)	Drug Target	PDB ID	RMSD_ave_ (Å)	RMSD_max_ (Å)	Log (Hit Rate)
ACE	1uze			0.60	HIVRT	1vrt			1.66
	1o86	0.17	0.37	0.45		1rt1	1.51	2.45	1.75
	1uzf	0.35	0.79	0.69		1c1c^a^	1.88	3.12	1.61
Alr2	1ah0			1.42		1rth^a^	1.62	2.28	1.61
	1ah3^a^	1.06	3.19	1.27	HMGR	1hw8			1.39
	2acr^a^^,^ b	0.88	1.72	1.10		1hwk	0.61	1.49	1.31
CDK2	1aq1^a^			1.32	NA	1a4g			0.57
	1buh^a^^,^ b	1.77	3.20	1.44		1a4q^a^	0.48	2.11	0.52
	1dm2^a^	1.75	4.49	1.62		1nsc^a^	0.34	1.49	0.52
COX-2	1cvu			1.51	P38 MAPK	1a9u			1.00
	1cx2^a^	1.24	3.78	1.53		1kv1	3.84	10.41	1.16
	3pgh^a^	1.11	3.96	1.64		1kv2	3.54	11.26	1.61
DHFR	3dfr			1.01	PDE5	1xoz			1.18
	6dfr^b^	1.47	1.96	1.02		1xp0	0.79	2.23	1.24
ER	1l2i			1.69	PPARg	1fm6			1.46
	3ert^a^	2.61	4.47	1.55		1fm9^a^	1.47	4.64	1.62
	1err^a^	2.01	4.39	1.61		2prg^a^	0.71	1.27	1.43
Fxa	1f0r			1.64	Thrombin	1ba8			1.53
	1fjs	1.09	2.57	1.59		1hgt^b^	0.69	1.85	1.55
	1ksn^a^	0.67	1.65	1.59	TK	1kim^a^			1.58
	1xka^a^	1.27	2.46	1.56		1ki4^a^	1.78	2.90	1.40

RMSD_ave_ was defined as the sidechain RMSD based on binding site residues within a cutoff distance of 4.5 Å from crystallographic ligands; RMSD_max_ is defined as the largest sidechain RMSD value among all the binding site residues.

a Structures used in the induced fit docking dataset of Sherman et al. [35].

b Apo structure, the rest are all holo structures.

ACE, angiotensin-converting enzyme; ALR2, aldose reductase; CDK2, cyclin-dependent kinase 2; COX-2, cyclooxygenase-2; DHFR, dihydrofolate reductase; ER, estrogen receptor; FXa, factor Xa; HIVRT, HIV reverse transcriptase; HMGR, hydroxymethylglutaryl-CoA reductase; NA, neuraminidase; P38 MAPK, P38 mitogen activated protein kinase; PDE5, phosphodiesterase 5; PPARg, peroxisome proliferator activated receptor gamma; TK, thymidine kinase.

All of these targets are predicted to be druggable based on the cutoff of 0.36 used above. Most of the targets have much higher druggability scores (>1.0), regardless of which crystal structure was used. The lowest druggability scores of 0.45 and 0.52 were calculated for angiotensin-converting enzyme (ACE, 1o86) and neuraminidase (NA, 1a4g), respectively. These binding sites are highly charged. It has been argued elsewhere that such binding sites are in fact less druggable than more hydrophobic binding sites [26]. Nevertheless, it is encouraging that our fragment virtual screening method is capable of assigning a reasonable druggability score to highly charged but druggable binding sites.

The histograms of energy scores (Figure S2) and the druggability scores calculated from them do change using different crystal structures, as expected, but in most cases the changes are remarkably small. The largest variation in the virtual fragment screening results between different crystal structures is seen for P38 MAPK, which also displays some of the largest conformational changes: the largest movement of a binding site side chain (RMSD_max._) is more than 10 Å, and the average movement of binding site side chains (RMSD_ave._) is ∼4 Å. In the other cases, where conformational changes in the binding site are relatively small (RMSD_max._<3 Å), the druggability scores vary only slightly, including for the cases where ligand-free structures were available (Alr2, CDK2, DHFR and thrombin). As shown below, the results on PPI sites show a much more striking dependence on conformational states.

### Druggability Assessment for Protein-Protein Interaction Sites

Protein-protein interactions are central to many biological processes, and therefore represent an important class of molecular targets for developing therapeutic agents. However, PPIs have been historically considered to be difficult targets for small molecular inhibitors due to the lack of suitable binding pockets to accommodate drug-like molecules. In addition, the binding interfaces in PPIs are generally highly conformationally flexible. Nevertheless, important progress has been made in discovering small molecular inhibitors of several important protein-protein interaction targets, such as MDM2-p53, BCL-X_L_-BAK and IL2-ILR [3], [36]. Therefore, it is desirable to computationally evaluate the druggability of PPIs, and identify specific druggable conformations.

Among the PPI sites studied here, high-affinity small molecule ligands have been found for IL-2, MDM2, BCL-X_L_, and HPV E2. MDM2 and BCL-X_L_ were correctly predicted to be druggable regardless of which crystal structure was used. By contrast, IL-2 and HPV E2 were correctly predicted to be druggable only when using the co-crystal structure with a small molecule inhibitor, and not when using a crystal structure with a peptide or protein bound. To date, only micromolar binders have been discovered for TNF and ZipA. ZipA was predicted to be non-druggable using both structures, while TNF was predicted to be druggable.

**Figure 3:**
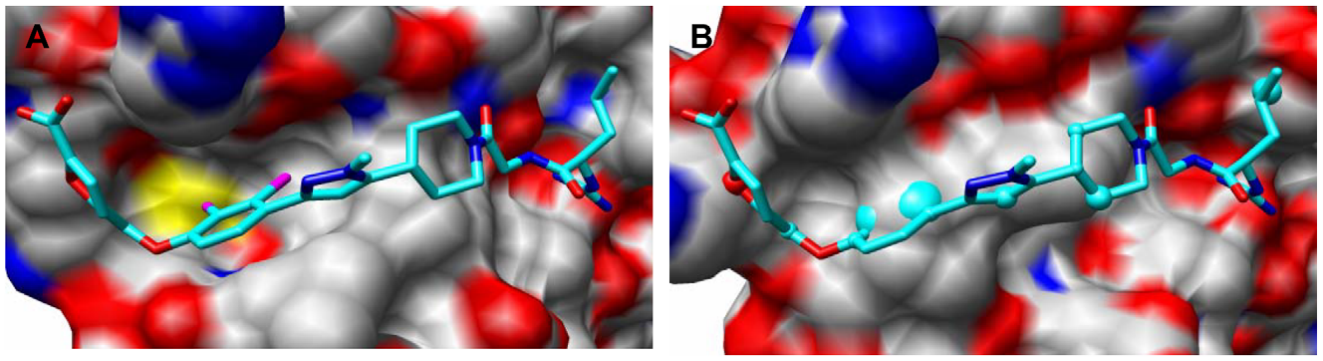
Conformational changes in IL-2. (A) IL-2 holo conformation bound with the co-crystallized ligand FRH (1py2). (B) The same ligand is superimposed on the apo conformation of the protein (1z92), highlighting the conformational changes. Molecular images were generated with UCSF Chimera [42].

In general, the structural variation among different structures of PPI targets (Table 3) is larger than in receptors or enzymes (Table 2). For example, as illustrated in Figure 3, a remarkable conformation change occurs upon ligand binding in IL-2. Clearly, the druggability score varies for different protein conformations [37], especially for IL-2, MDM2, and HPV E2 (Table 3 and Figure S3).

**Table 3:** Druggability scores calculated for 6 targets involved in protein-protein interactions.

PPI Target	PDB ID	RMSD_ave._ (Å )	RMSD_max._ (Å )	Log (Hit Rate)	PPI Target	PDB ID	RMSD_ave._ (Å )	RMSD_max._ (Å )	Log (Hit Rate)
IL-2	1z92^a^			0.13	MDM2	1ycr^a^			0.45
	1py2	2.59	5.80	0.62		1rv1	1.82	3.32	0.92
	1m48	2.51	4.57	0.62		1t4e	1.57	2.91	0.66
BCL-X_L_	2bzw^a^			1.04	HPV E2	1tue^a^			−0.24
	2yxj	2.54	6.16	0.84		1r6n	2.80	4.32	1.02
TNF	1tnf^a^			0.95	ZipA	1f47^a^			−0.02
	2az5	2.90	5.65	0.96		1y2f	0.59	1.26	−0.10

a Reference structure bound with protein or peptide substrate; the remaining structures contain small molecule ligands.

### Two Case Studies

**Figure 4:**
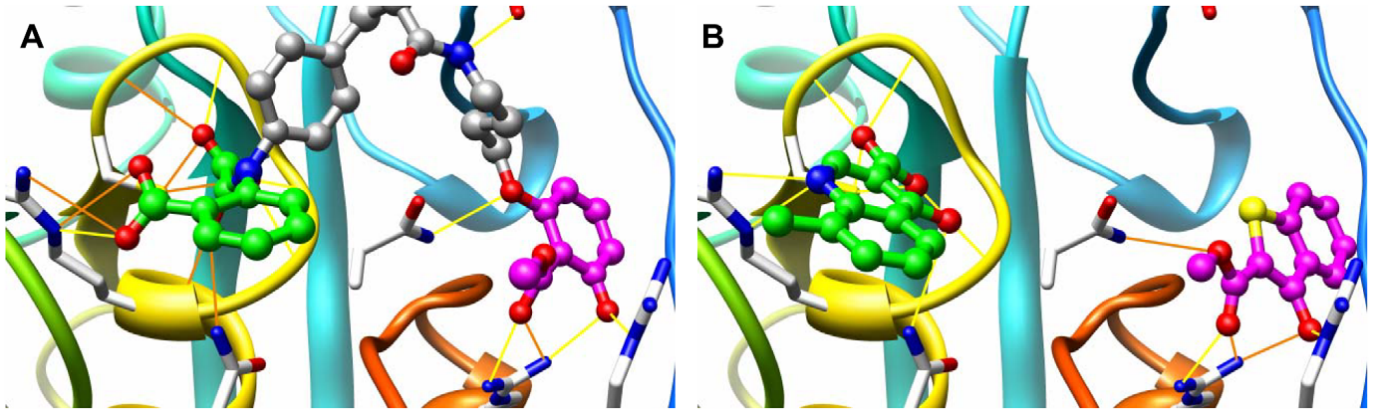
Top ranked fragments from the virtual screen mimic portions of a known potent PTP1B inhibitor. (A) A co-crystallized ligand (stick) is shown bound to PTP1B (1ph0), and extends across both the catalytic and non-catalytic sites. The key hydrogen bonding interactions between the ligands and the binding site residues are illustrated with yellow lines. Different portions of the ligand are colored for comparison with the fragments in (B). (B) Two high-ranking fragments from virtual screening. One predicted heterocyclic carboxylic acid (carbon atoms colored green, rank 49) is shown bound to the PTP1B catalytic site, and one neutral methyl salicylate hit (carbon atoms colored magenta, rank 59) is shown bound to the non-catalytic site. Molecular images were generated with UCSF Chimera [42].

Here we examine in more depth two case studies, focusing on the chemical composition and binding modes of top-ranked fragments. The first case study is protein tyrosine phosphatase 1B (PTP1B). The PTP1B catalytic site and vicinal non-catalytic site are surface-exposed, highly hydrophilic, and recognize charged phosphotyrosine (pTyr) residue or pTyr mimetics (Figure 4A). Characterizing the binding properties of such sites is particularly challenging because the binding sites contain numerous polar and charged groups, and ligand binding is a complex tradeoff between forming favorable electrostatic interactions and the cost of desolvation. NMR-based screening results suggested that the catalytic site is highly druggable while the non-catalytic site is non-druggable. The Hajduk et al. empirical model assigned the PTP1B catalytic site as moderately druggable [27], while the computational solvent mapping technique predicted this site to be non-druggable [23]. Encouragingly, our virtual screening method correctly classifies the catalytic and non-catalytic sites as druggable and non-druggable, respectively. It even reproduces the relative hit rate observed in the NMR experiment (∼10 times higher for the catalytic site), although this may be a fortuitous result. Appropriately, the high-ranking fragment hits identified for the PTP1B catalytic site are dominated by heterocyclic carboxylic acids, and hits for the non-catalytic site contain many neutral methyl salicylate moieties (Figure 4B), which is consistent with previously identified fragments from experimental screening studies (chemical structures are shown in Figure S4) [38], [39].

**Figure 5:**
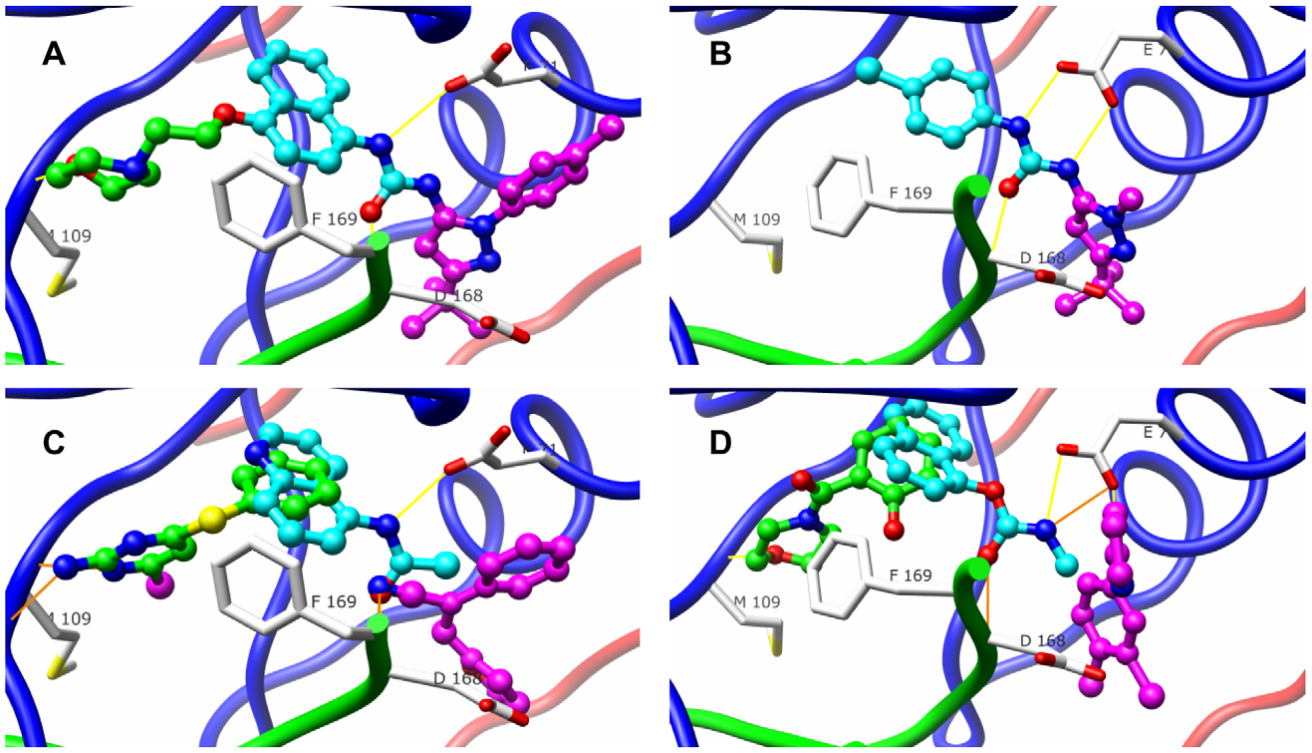
Top ranked fragments from the virtual screen mimic portions of known inhibitors of p38 MAP kinase. (A) The high affinity inhibitor BIRB796 (stick) is shown bound to p38 MAP kinase (1kv2). Key hydrogen bonding interactions—between the morpholino group and the main chain amide of residue Met109, the urea group and the side chain carboxylate group of conserved residue Glu71, and the main chain amide of residue Asp168—are illustrated with yellow lines. Portions of the ligand are colored for comparison with fragments in panels (C) and (D). (B) A low-affinity ligand BMU (stick) is shown bound to the allosteric pocket (1kv1). (C) Three partially overlapping top fragment hits (stick) identified from virtual screening against the 1kv2 structure are shown: a pyridinyl-imidazole type of fragment (carbon atoms colored green, rank 155 in the virtual screen) bound to the ATP binding pocket; a urea-like moiety on a substituted naphthyl ring (carbon atoms colored cyan, rank 55) interacting with the Glu71 sidechain and the lipophilic pocket; and a substituted heterocyclic ring (carbon atoms colored magenta, rank 165) deeply buried into the allosteric binding pocket. (D) The overlap of three top scored fragment hits identified from virtual screening against the 1kv1 structure (ranks 6, 136 and 210). Molecular images were generated with UCSF Chimera [42].

The second case study is P38 MAPK, which was chosen due to its significant structural flexibility, where a new allosteric binding site spatially distinct from the ATP catalytic pocket is induced upon binding to a diaryl urea type of inhibitor. We focus on the crystal structure (1kv2) bound with most potent MAPK inhibitor, BIRB796 (Figure 5A), and the crystal structure (1kv1) bound with a micomolar diaryl urea type of inhibitor (Figure 5B). BIRB796 binds potently by forming strong interactions to both the ATP binding pocket and the allosteric site, including the crucial hydrogen bonding interaction between the morpholino group and the main chain amide of residue Met109; the hydrophobic interaction between its naphthyl moiety and the lipophilic pocket formed by the side chains of Lys53, Leu75, Ile84, Leu104 and Thr106; the hydrogen bonds between the urea group and the side chain carboxylate group of residue Glu71 and the main chain amide of residue Asp168; and the hydrophobic interactions between its tolyl and t-butyl groups on the pyrazole ring and the allosteric pocket [40]. The top scoring fragment hits occupy different binding regions. For example, pyridinyl-imidazole scaffolds are predicted to target the ATP binding pocket, while urea-like moieties on substituted naphthyl rings establishing interactions with the Glu71 sidechain and the lipophilic pocket, and substituted heterocyclic rings bury deeply into the allosteric binding pocket (Figure 5C). It is notable that the predicted fragment bound to the ATP binding pocket structurally resembles an aminopyridine type of fragment identified by fragment-based crystallography screening (chemical structures are shown in Figure S5) [39]. The results using the crystal structure bound with a micomolar diaryl urea type of inhibitor are similar, and high-ranking fragment hits mimic binding interactions seen for the picomolar inhibitor (Figure 5D).

In summary, we have developed an *in silico* fragment screening method, analogous to an NMR-based screening method that was previously shown to be effective at assessing the druggability of the binding sites. This approach does not require fitting any physicochemical parameters derived from protein binding pocket. We performed a large scale assessment on a total of 152 protein binding sites. We demonstrated that the hit rate calculated for 21 binding sites using our approach correlates with the hit rate measured experimentally from NMR-based screening method, and that the method could successfully distinguish known druggable and non-druggable targets. We are investigating extensions of the method to identifying druggable conformations of flexible binding sites for molecular docking, and suggesting strategies for growing or joining initial fragment hits to obtain more potent inhibitors.

## Methods

### Dataset Selection

24 binding sites (Table 1) were chosen from the training dataset of Hajduk et al. [27] to develop our physics-based druggability prediction model. A total of 28 binding sites were experimentally investigated via the NMR-based fragment screening approach. However, structural information for CMPK_other_, E2–31_other_ and Survivin_other_ sites are not publicly available, and the crystal structure of MurA is fosfomycin-covalent modified, so these 4 binding sites were excluded in our study. The external dataset defined by Hajduk et al. [27] contains 72 proteins, of which 35 binding sites were assigned as druggable and the remaining 37 sites as non-druggable, based on whether high-affinity druglike binders could be found in the literature. We have used all 72 binding sites (Table S1) to assess our prediction on new targets. For both the training and the external datasets, the same protein structures were used as in the Hajduk et al. study unless it was not reported (i.e. Bcl-X_L_, CMPK, E2–31, PAK4 and SARS-RNA site).

To supplement these targets, we selected 15 well-known drug targets from the DUD dataset [41] as true positives, and two or more crystal structures were chosen for each target (Table 2). Many of these structures were also used by Sherman et al. for testing a strategy for docking against flexible binding sites [35]. We use these flexible binding sites to evaluate the consequence of structural flexibility in our druggability calculation. Finally, six protein-protein interaction (PPI) targets [3] (Table 3) were chosen from the recent literature.

To quantitate the flexibility of ligand binding sites, the crystal structures for each target were superimposed using Chimera [42], and root mean squared distance (RMSD) values were calculated for each binding site residue or a combination of several binding site residues. Residues that could not be aligned, as well as those with missing atoms, were ignored during RMSD calculations.

### Fragment Library Preparation

The diverse set of fragments was selected from the fragment-like subset of the ZINC database (version 6, December 2005) [43]. This subset contains 49,134 compounds with relatively low molecular weight (MW ≤ 250), few rotatable bonds (RB < 3), low hydrophobicity (−2 < log P < 3), and weak hydrogen bonding potentials (HB_donor_ <3 and HB_acceptor_ <6). We also eliminated fragments with more than 15 heavy atoms, based on the previous observation that maximal binding free energy increases more slowly for ligands containing more than 15 heavy atoms [44]. This filter reduced the library size to 32,717 molecules. Finally, we performed structural similarity analysis to reduce redundancy. Feature key fingerprints were calculated using CACTVS [45] and the fingerprint-based similarity analysis was performed with a modified version of the program SUBSET [46]. Representative structures were selected for each structural cluster with Tanimoto coefficient (Tc) less than 0.9 to other clusters. This further reduced the library to 11,129 diverse molecules. To assess any potential bias resulting from the diversity-based filtering, 32,717 ZINC fragment-like compounds were used to redo the screening for the Hajduk et al. training dataset. The computed energy distributions are very similar (data not shown).

### Computational Druggability Assessment Protocol

**Figure 6:**
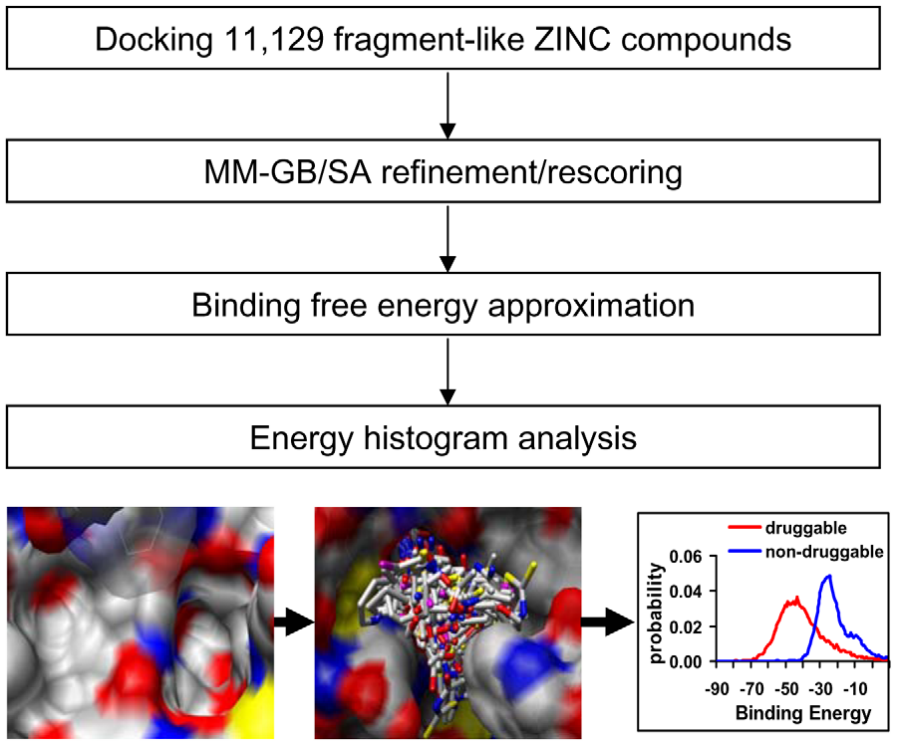
A virtual fragment screening protocol for druggability assessment.

The detailed virtual screening protocol was published elsewhere [28], [47]; here, a brief overview is presented (Figure 6). Our scoring method consists of two steps: predicting the binding poses of ligands using a docking program, and then refining and rescoring those protein-ligand complexes using a more computationally intensive molecular-mechanics based energy function. This protocol uses a high-throughput docking program to initially orient and score the ZINC fragment-like compounds in the binding site, and subjects the best single docking pose for each docked compound to a rescoring stage in which the ligand is energy minimized and the binding affinity is estimated using an all-atom molecular mechanics force field combined with an implicit solvent model. Most of the labor-intensive, manual steps during the docking and rescoring stages were automated for large scale application here.

The program DOCK 3.5.54 was used to dock the fragment database into the protein binding site [48], [49]. A maximum of 120 matching spheres were used to ensure adequate ligand sampling within large binding surfaces like protein-protein interfaces. Ligand conformations were scored based on the docking total energy (E_tot_ = E_ele_ + E_vdw_ − ΔG_lig-solv_), which was the sum of electrostatic (E_ele_) and van der Waals (E_vdw_) interaction energies corrected by the ligand partial desolvation energy (ΔG_lig-solv_) [49]. Final energies were computed after rigid-body minimization. Then, a single docking pose with the best total energy score was saved for each docked molecule for the next stage of scoring.

The docked protein-ligand complex and ligand were then submitted to multi-scale Truncated Newton energy minimization in all-atom OPLS force field and Generalized Born (GB) solvent using the Protein Local Optimization Program (PLOP) [50], [51], [52]. The molecular mechanics forces are divided into short-range (bond, angle, torsion, and local non-bonded) and long-range components, with the long-range forces updated only intermittently. The algorithm was also optimized for minimizations with GB solvent that increases the computational expense by only a factor of ∼3 relative to the vacuum. Thus, this scoring approach accounts for accurate and efficient calculations of ligand-protein interaction energies, the ligand/receptor desolvation, and to a lesser extent, ligand strain energies. In this work, the protein was kept rigid during ligand-protein complex minimization to reduce the computational expense. The binding energy (E_bind_ = E^R*L^ − E^L^ − E^R^) was calculated by subtracting the energies of the optimized free ligand in solution (E^L^) and the free protein in solution (E^R^) from the optimized ligand-protein complex's energy in solution (E^R*L^). The van der Waals energy component was empirically scaled by a factor of 2 as we suggested previously [28].

To compute a “hit rate” for the *in silico* screening, we chose an energy cutoff value empirically to maximally differentiate druggable and non-druggable binding sites. The “druggability score” in this work is defined as log(hit rate). At the present time, docking scoring functions cannot robustly reproduce absolute binding affinities in realistic applications. Although useful for rank-ordering compounds [47], the molecular-mechanics based scoring function used here also cannot be interpreted in terms of absolute binding affinities, in part because entropy losses are not computed. The cutoff chosen (−40 kcal/mol) was based primarily on visual inspection of the energy distributions for the 13 druggable binding sites and 11 non-druggable sites in the Hajduk et al. training data set. The effect of varying the cutoff is explored with respect to differentiating between druggable and non-druggable binding sites (Figure S6). Interestingly, the correlation between the docking screening hit rates and the NMR screening results is relatively insensitive to the value of the energy cutoff within a certain range (from −40 to −34 kcal/mol). Unless stated otherwise, the results below use an energy cutoff of −40 kcal/mol for computing the in silico “hit rate”.

## Supporting Information

Table S1 Targets, binding sites, available ligand binding information, and hit rate data predicted by two different computational models.
(0.19 MB DOC)

Figure S1 Energy histograms from docking 11,129 ZINC fragment-like compounds against 24 binding sites previously studied by NMR-based fragment screening. Color code is defined using the NMR druggability score: druggable (green): log (Hit Rate) >−1.0, and non-druggable (red): log (Hit Rate) ≤−1.0.
(1.56 MB TIF)

Figure S2 Histograms of energy scores from the virtual fragment screening method for 15 well-known drug targets. ACE, angiotensin-converting enzyme; ALR2, aldose reductase; CDK2, cyclin-dependent kinase 2; COX-2, cyclooxygenase-2; DHFR, dihydrofolate reductase; ER, estrogen receptor; FXa, factor Xa; HIVRT, HIV reverse transcriptase; HMGR, hydroxymethylglutaryl-CoA reductase; NA, neuraminidase; P38 MAPK, P38 mitogen activated protein kinase; PDE5, phosphodiesterase 5; PPARg, peroxisome proliferator activated receptor gamma; TK, thymidine kinase.
(1.59 MB TIF)

Figure S3 Energy histograms of docking 11,129 ZINC fragment-like compounds against 6 targets involved in protein-protein interactions. Color code is defined as druggable (green) and non-druggable (red).
(1.03 MB TIF)

Figure S4 Chemical structures of a ligand co-crystallized with PTP1B (1ph0), binders identified in experimental screening, and high-ranking fragment hits identified from virtual fragment screening (fragments bound to the catalytic site are colored in green and to the non-catalytic site in magenta).
(1.01 MB TIF)

Figure S5 Chemical structures of a ligand co-crystallized with P38 MAPK (1kv2), binders identified in experimental screening, and high-ranking fragment hits identified from virtual fragment screening using two different crystal structures, 1kv2 and 1kv1 (fragments bound to ATP site colored in green, lipophilic pocket colored in cyan, and allosteric site in magenta).
(1.06 MB TIF)

Figure S6 The correlation between the virtual fragment screening hit rates and the NMR screening results, using different energy cut-offs for defining the fragment-like compounds as “hits” in the virtual screen.
(0.78 MB TIF)
