# Supplementary material for: Binding-Site Assessment by Virtual Fragment Screening
Source: PLoS One. 2010 Apr 9;5(4):e10109. doi: 10.1371/journal.pone.0010109 (PMC2852417; doi:10.1371/journal.pone.0010109)
Supplement: Table S1 — Targets, binding sites, available ligand binding information, and hit rate data predicted by two different computational models. (0.19 MB DOC) [file pone.0010109.s001.doc]

Table S1. Targets, binding sites, available ligand binding information, and hit rate data predicted by two different computational models.

| **No** | **PDB ID** | **Protein** | **Ligand** | **High affinity, drug-like ligand?** ^a^ | **Best Kd (nM)**^a^ | **Hajduk et al. Predicted Score** ^a^ | **Virtual fragment screening Score** |
| --- | --- | --- | --- | --- | --- | --- | --- |
| 1 | 1ldg | Lactate dehydrogenase | NAD | Yes | 30 | 0.288 | 1.377 |
| 2 | 1lyb | Cathepsin D | Pepstatin | Yes | 250 | 0.053 | 0.799 |
| 3 | 1lpm | Lipase | MPA | Yes | 100 | -0.030 | 0.543 |
| 4 | 1eno | ENOYL ACP reductase | NAD | Yes | 50 | -0.065 | 1.534 |
| 5 | 1mmp | Matrilysin | Inhibitor | Yes | 200 | -0.103 | 0.612 |
| 6 | 1bit | Trypsin | Benzamidine | Yes | 8 | -0.108 | 1.379 |
| 7 | 1fds | 11b-HSD | Estradiol | Yes | 20 | -0.113 | 1.277 |
| 8 | 1b6a | MetAP2 | TNP | Yes | 33 | -0.218 | 1.542 |
| 9 | 1aha | Mormorcharin | Adenine | No |  | -0.327 | -0.254 |
| 10 | 1fmc | 7a-HSD | Cholic acid analog | Yes | 75 | -0.333 | 1.288 |
| 11 | 1n83 | ROR-LBD | Cholesterol | Yes | 280 | -0.343 | 1.571 |
| 12 | 1gar | Transformylase | Inhibitor | Yes | 15 | -0.358 | 0.945 |
| 13 | 1lcb | Thymidilate synthase | TMP | Yes | 250 | -0.360 | 0.595 |
| 14 | 1add | Adenosine deaminase | Deazaadenosine | Yes | 50 | -0.413 | 0.804 |
| 15 | 1hmr | FABP ^b^ | Elaidic acid | No | >1000 | -0.458 | 1.302 |
| 16 | 1nt4 | Glucose-1-phosphatase ^c^ | Glucose-1-phosphate | No |  | -0.498 | 0.358 |
| 17 | 1ccg | Cytochrome c peroxidase | Imidazole | No |  | -0.504 | -2.046 |
| 18 | 1daa | D-amino-acid-transferase | Pyridoxal-5'-phosphate | No |  | -0.513 | -0.005 |
| 19 | 1pso | Pepsin | Pepstatin | Yes | 39 | -0.514 | 0.813 |
| 20 | 1dhr | Dihydropteridine reductase ^b^ | NADH | No |  | -0.515 | 0.891 |
| 21 | 1drv | DHPR | NADH | Yes | 100 | -0.531 | 0.479 |
| 22 | 1lst | LAO-binding-protein | Lys | No |  | -0.562 | -0.189 |
| 23 | 1aec | Actinidin | E64 | Yes | 19 | -0.563 | 0.932 |
| 24 | 1q5h | DUTP pyrophosphatase | DUD | No |  | -0.593 | -0.724 |
| 25 | 1ahh | a-hydroxysteroid dehydrogenase | NAD | Yes | 9 | -0.631 | 1.502 |
| 26 | 1ose | a-amylase ^c^ | Acarbose | No |  | -0.640 | 0.695 |
| 27 | 1bls | b-lactamase | IPP | Yes | 1 | -0.671 | 0.891 |
| 28 | 1drh | Dihydrofolate reductase | NADP | Yes | 23 | -0.693 | 1.129 |
| 29 | 1epb | RABP | RA | Yes | 300 | -0.709 | 1.236 |
| 30 | 1bmd | Malate dehydrogenase | NADH | No | 560 | -0.737 | 0.300 |
| 31 | 1cbx | Carboxypeptidase | Inhibitor | Yes | 55 | -0.762 | 1.017 |
| 32 | 1fsl | Leghemoglobin | Nicotinate | No |  | -0.771 | -0.107 |
| 33 | 1aer | Exotoxin | TAD | Yes | 87 | -0.776 | 1.272 |
| 34 | 1rne | Renin | Inhibitor | Yes | 0.8 | -0.780 | 0.842 |
| 35 | 1ndc | Nucleoside diphosphate kinase | TYD | Yes | 15 | -0.807 | 0.525 |
| 36 | 1l7m | Phosphoserine-phosphatase | MSE | No | >1000 | -0.808 | 0.358 |
| 37 | 1rcf | Flavodoxin | FMN | No |  | -0.857 | 0.665 |
| 38 | 1qcf | HCK | Inhibitor | Yes | <100 | -0.916 | 1.487 |
| 39 | 1hmy | DNA methyltransferase | SAM | Yes | 70 | -0.933 | 1.192 |
| 40 | 1ads | Aldose reductase | NADPH | Yes | 13 | -0.954 | 1.486 |
| 41 | 1cen | Cellulase ^c^ | Cellohexaose | No | 400 | -0.975 | 0.265 |
| 42 | 1igb | Aminopeptidase | IPO | Yes | 66 | -1.010 | 1.201 |
| 43 | 1fmb | EIAV-protease | Inhibitor | Yes |  | -1.032 | 1.171 |
| 44 | 1awb | Inositol-monophosphatase ^b^ | IPD | No | >1000 | -1.040 | 1.245 |
| 45 | 1dds | Dihydrofolate reductase | Methotrexate | Yes | 5 | -1.165 | 0.794 |
| 46 | 1eus | Sialidase | Neuraminic acid analog | Yes | 0.8 | -1.201 | 0.684 |
| 47 | 1gym | PI-phospholipase | Inositol-analog | No | >1000 | -1.204 | -0.064 |
| 48 | 1lta | Enterotoxin ^c^ | Galactose | No |  | -1.221 | 0.209 |
| 49 | 1pfk | Phosphofructo kinase | FBP | No |  | -1.316 | -1.201 |
| 50 | 1ptr | PKC-delta | Phorbol | Yes | <10 | -1.334 | 0.025 |
| 51 | 1aco | Aconitase | Transaconitate | No |  | -1.336 | -0.528 |
| 52 | 121p | Ras-p21 | GTP | Yes | 2 | -1.362 | 1.284 |
| 53 | 1gky | Guanylate kinase ^d^ | GMP | No |  | -1.406 | 1.531 |
| 54 | 1qca | Chloramphenicol-acetyltransferase | Fusidic acid | No |  | -1.439 | 0.071 |
| 55 | 1gfi | Guanine nucleotide-binding protein ^d^ | GDP | No |  | -1.480 | 1.737 |
| 56 | 1dek | Deoxynucleoside-monophosphate-kinase ^d^ | GMP | No |  | -1.503 | 1.560 |
| 57 | 1bkm | Src transforming protein | 1C5 | No |  | -1.561 | -0.005 |
| 58 | 1dog | Glucoamylase ^c^ | 1-deoxynojirimycin | No | >1000 | -1.602 | 0.489 |
| 59 | 1rrg | ADP ribosylatin-factor | GDP | No |  | -1.602 | -0.078 |
| 60 | 1aor | Aldehyde-ferredoxin-oxidoreductase | Molbdopterin | No |  | -1.608 | -2.046 |
| 61 | 1glg | Galactose binding protein ^c^ | Galactose | No |  | -1.630 | 0.278 |
| 62 | 1ctu | Cytidine deaminase | Riboside | Yes | 15 | -1.632 | 1.274 |
| 63 | 1mrj | trichosanthin | Adenine | No |  | -1.744 | 0.721 |
| 64 | 1arc | Achromobacter protease | TCK | No |  | -2.000 | 0.751 |
| 65 | 1bwn | PH-domain | INS | No | >1000 | -2.000 | 0.519 |
| 66 | 1dap | Diaminopimelic acid dehydrogenase | NADP+ | No | >100,000 | -2.000 | 0.323 |
| 67 | 1dud | Deoxyuridine-nucleotide hydrolase | D-UDP | No |  | -2.000 | -0.254 |
| 68 | 1ed8 | Alkaline phosphatase | PO4 | No |  | -2.000 | -0.666 |
| 69 | 1gca | GBP ^c^ | Galactose | No |  | -2.000 | 0.452 |
| 70 | 1i9z | IP-phosphatase | 2IP | No | 230 | -2.000 | -0.870 |
| 71 | 1nv8 | Glutamine-methyltransferase | SAM | No |  | -2.000 | 0.843 |
| 72 | 1os5 | HCV- polymerase | Inhibitor | Yes | 45 | -2.000 | 1.079 |

^a^ from Reference[1]

^b^ Targets are potentially druggable based on our survey of the literature. #15: FABP (ref.[2,3]), #20: dihydropteridine reductase (ref.[4]), #44: inositol-monophosphatase (ref.[5]).

^c^ Sugar or sugar-analog binding proteins with no high-affinity drug-like binders reported, where our predicted druggability scores are generally moderate.

^d^ Nucleoside/nucleotide binding proteins that were predicted to be druggable in our calculations.

Refs: 1. Hajduk PJ, Huth JR, Fesik SW (2005) Druggability indices for protein targets derived from NMR-based screening data. J Med Chem 48: 2518-2525.

2. van Dongen MJ, Uppenberg J, Svensson S, Lundback T, Akerud T, et al. (2002) Structure-based screening as applied to human FABP4: a highly efficient alternative to HTS for hit generation. J Am Chem Soc 124: 11874-11880.

3. Furuhashi M, Tuncman G, Gorgun CZ, Makowski L, Atsumi G, et al. (2007) Treatment of diabetes and atherosclerosis by inhibiting fatty-acid-binding protein aP2. Nature 447: 959-965.

4. Abell CW, Shen RS, Gessner W, Brossi A (1984) Inhibition of dihydropteridine reductase by novel 1-methyl-4-phenyl-1,2,3,6-tetrahydropyridine analogs. Science 224: 405-407.

5. Zheng W, Brandish PE, Kolodin DG, Scolnick EM, Strulovici B (2004) High-throughput cell-based screening using scintillation proximity assay for the discovery of inositol phosphatase inhibitors. J Biomol Screen 9: 132-140.
