# Supplementary figures and images for: Binding-Site Assessment by Virtual Fragment Screening
Source: PLoS One. 2010 Apr 9;5(4):e10109. doi: 10.1371/journal.pone.0010109 (PMC2852417; doi:10.1371/journal.pone.0010109)

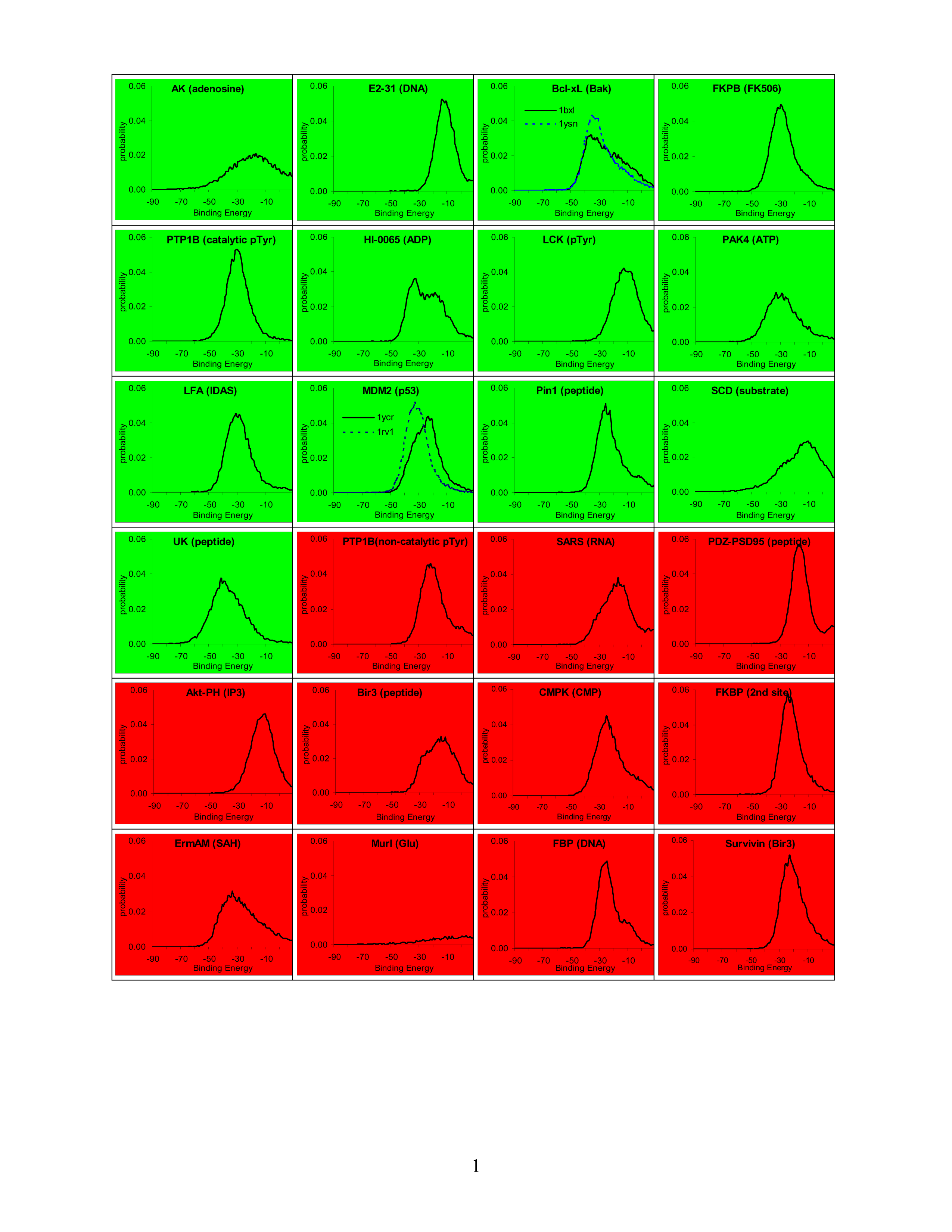

Supplement: Figure S1 — Energy histograms from docking 11,129 ZINC fragment-like compounds against 24 binding sites previously studied by NMR-based fragment screening. Color code is defined using the NMR druggability score: druggable (green): log (Hit Rate) >−1.0, and non-druggable (red): log (Hit Rate) ≤−1.0. (1.56 MB TIF) [file pone.0010109.s002.tif]

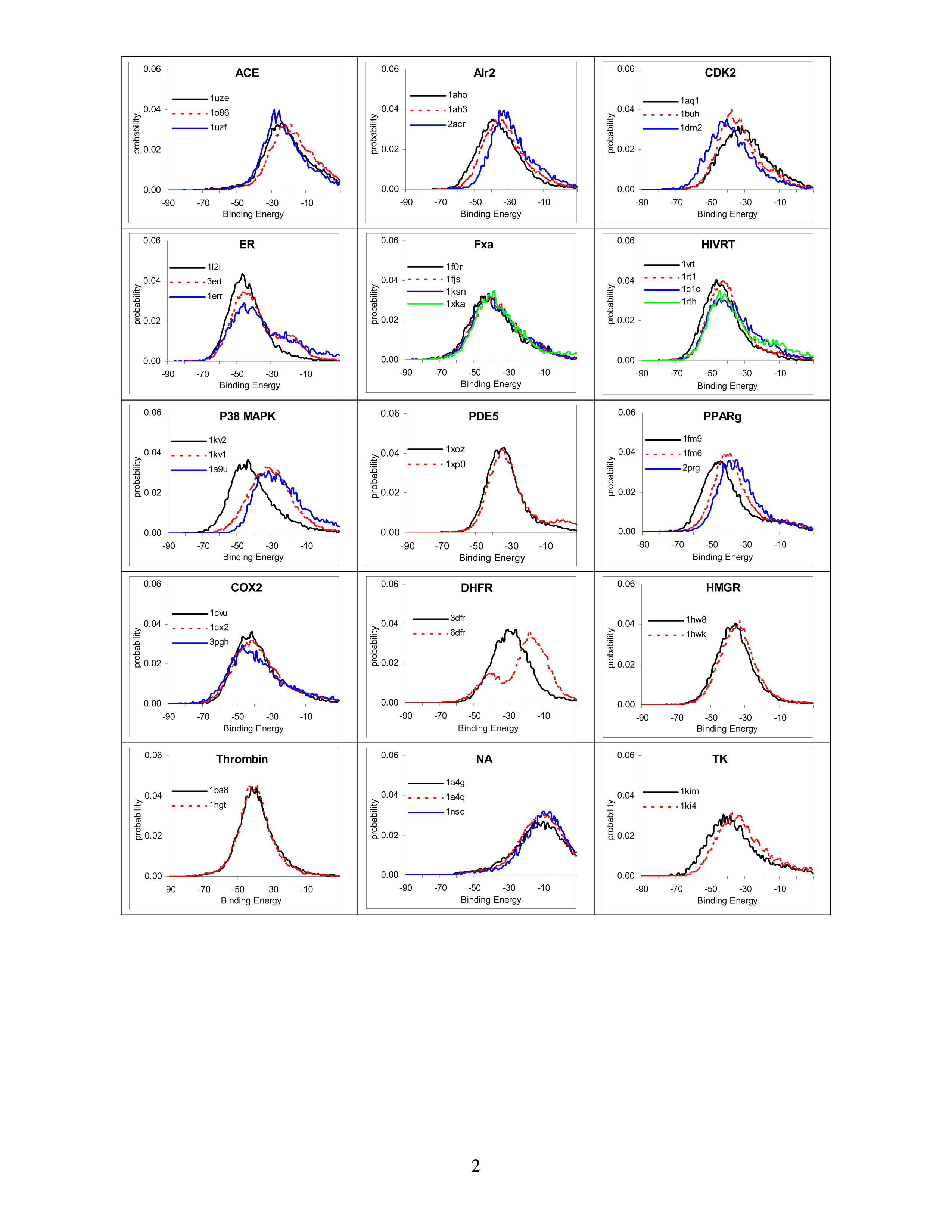

Supplement: Figure S2 — Histograms of energy scores from the virtual fragment screening method for 15 well-known drug targets. ACE, angiotensin-converting enzyme; ALR2, aldose reductase; CDK2, cyclin-dependent kinase 2; COX-2, cyclooxygenase-2; DHFR, dihydrofolate reductase; ER, estrogen receptor; FXa, factor Xa; HIVRT, HIV reverse transcriptase; HMGR, hydroxymethylglutaryl-CoA reductase; NA, neuraminidase; P38 MAPK, P38 mitogen activated protein kinase; PDE5, phosphodiesterase 5; PPARg, peroxisome proliferator activated receptor gamma; TK, thymidine kinase. (1.59 MB TIF) [file pone.0010109.s003.tif]

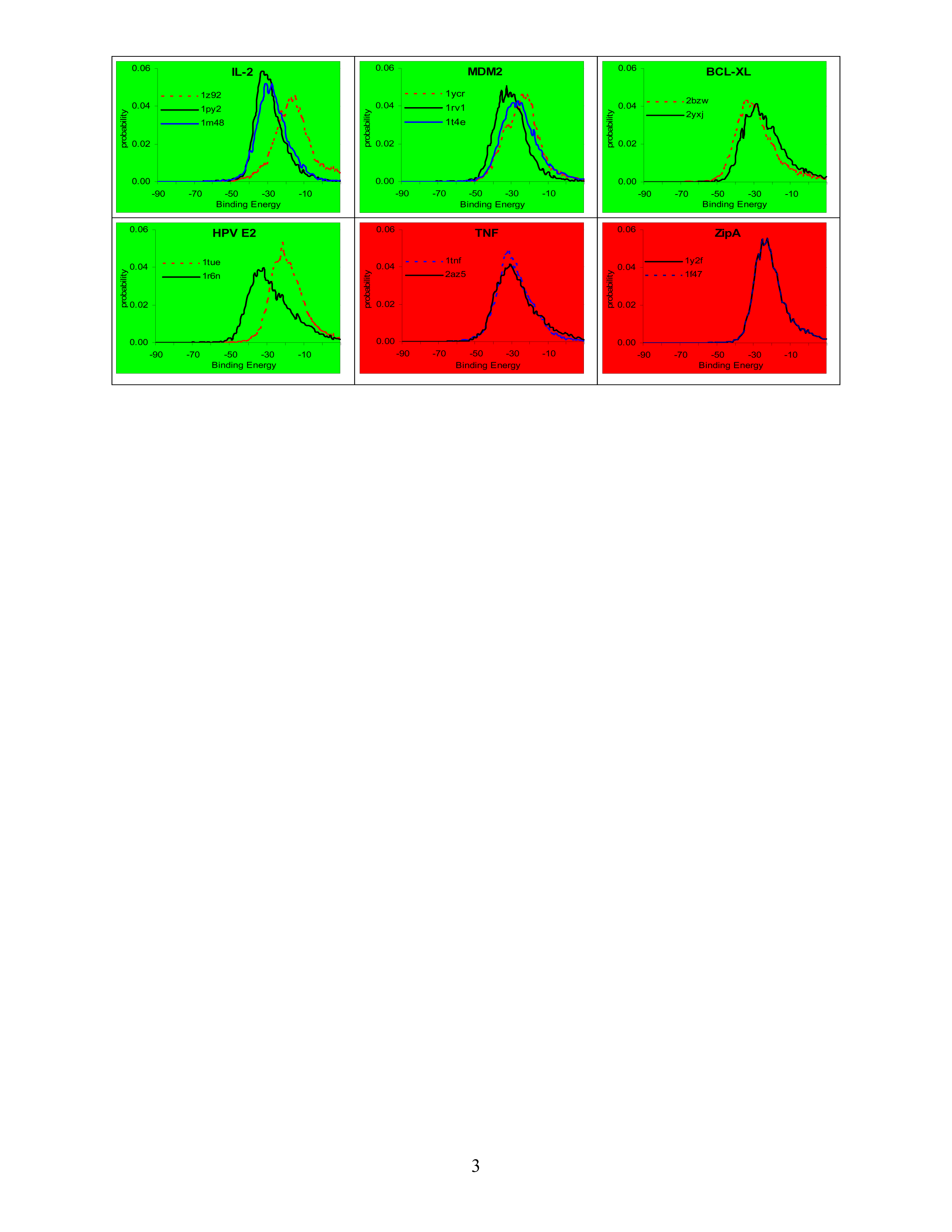

Supplement: Figure S3 — Energy histograms of docking 11,129 ZINC fragment-like compounds against 6 targets involved in protein-protein interactions. Color code is defined as druggable (green) and non-druggable (red). (1.03 MB TIF) [file pone.0010109.s004.tif]

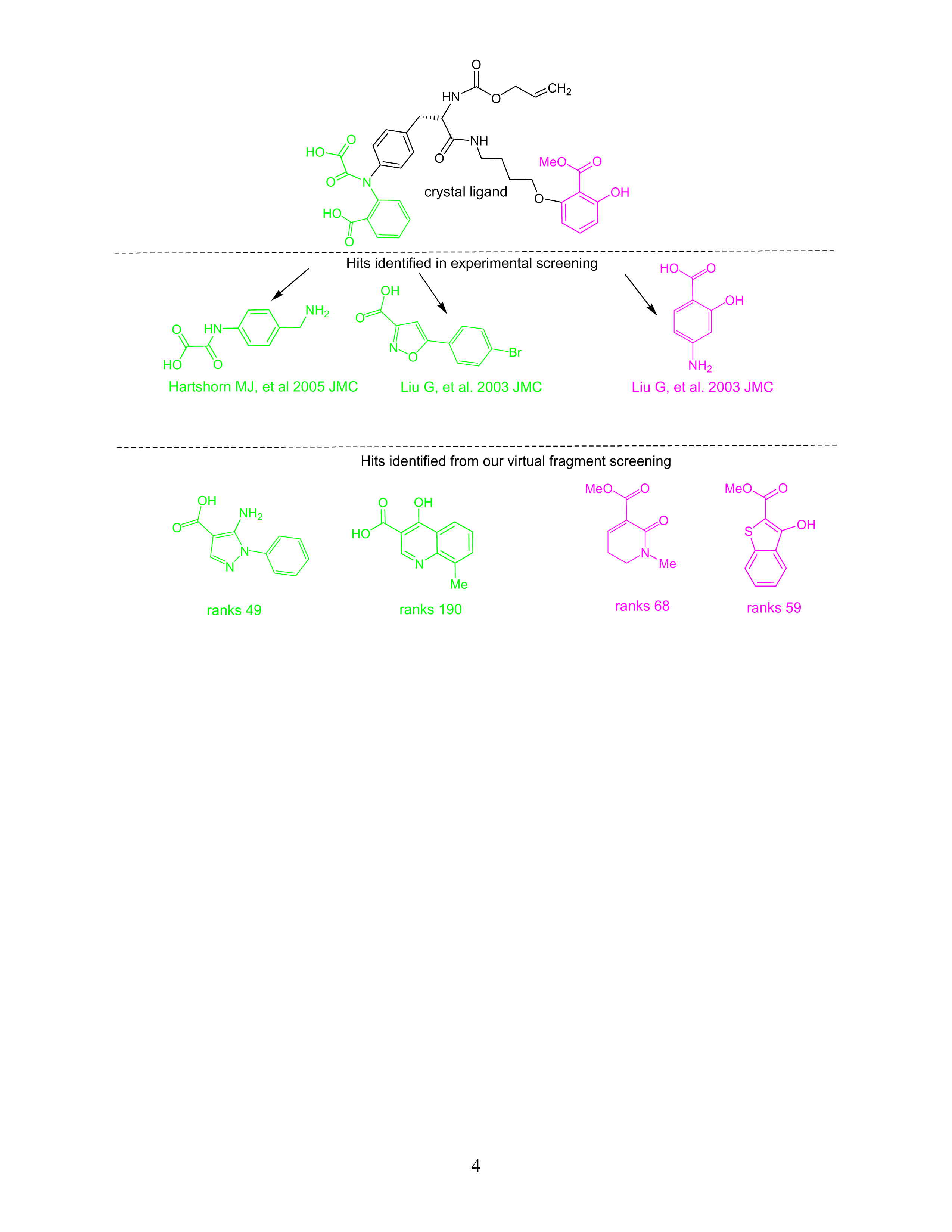

Supplement: Figure S4 — Chemical structures of a ligand co-crystallized with PTP1B (1ph0), binders identified in experimental screening, and high-ranking fragment hits identified from virtual fragment screening (fragments bound to the catalytic site are colored in green and to the non-catalytic site in magenta). (1.01 MB TIF) [file pone.0010109.s005.tif]

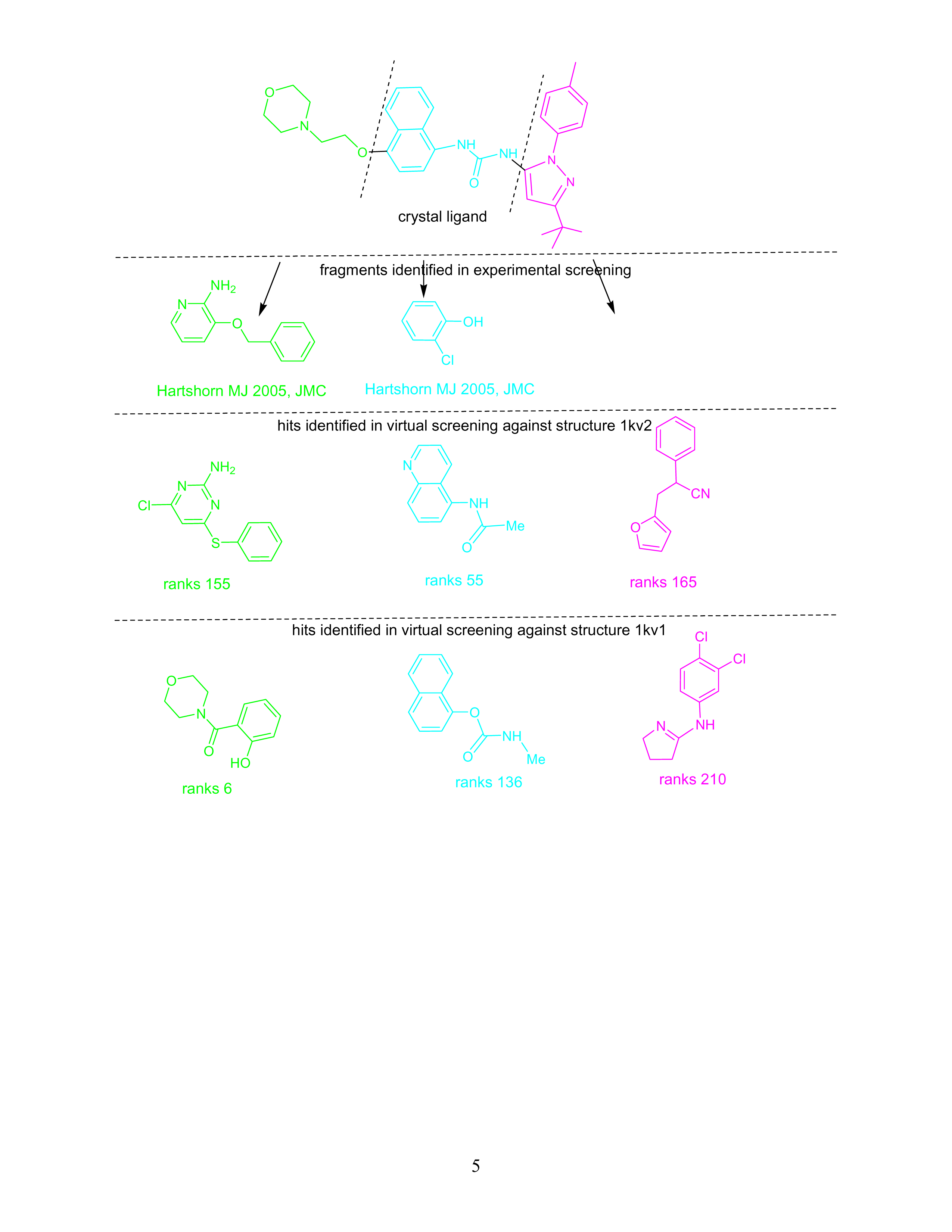

Supplement: Figure S5 — Chemical structures of a ligand co-crystallized with P38 MAPK (1kv2), binders identified in experimental screening, and high-ranking fragment hits identified from virtual fragment screening using two different crystal structures, 1kv2 and 1kv1 (fragments bound to ATP site colored in green, lipophilic pocket colored in cyan, and allosteric site in magenta). (1.06 MB TIF) [file pone.0010109.s006.tif]

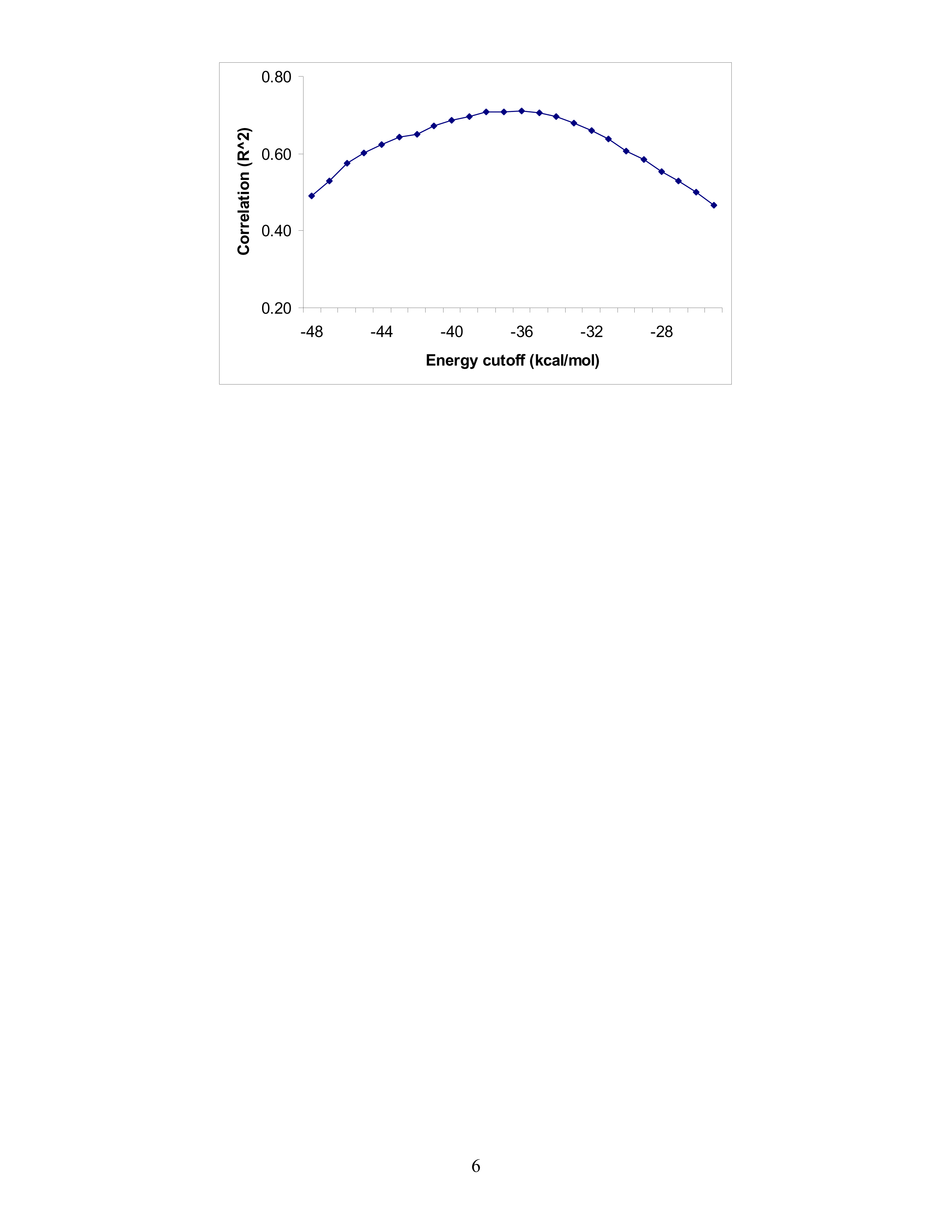

Supplement: Figure S6 — The correlation between the virtual fragment screening hit rates and the NMR screening results, using different energy cut-offs for defining the fragment-like compounds as “hits” in the virtual screen. (0.78 MB TIF) [file pone.0010109.s007.tif]
